# Control of Glycogen Content in Retina: Allosteric Regulation of Glycogen Synthase

**DOI:** 10.1371/journal.pone.0030822

**Published:** 2012-02-17

**Authors:** Ixchel Osorio-Paz, Gustavo Sánchez-Chávez, Rocío Salceda

**Affiliations:** División Neurociencias, Instituto de Fisiología Celular, Universidad Nacional Autónoma de México, México, Distrito Federal, Mexico; City of Hope National Medical Center and Beckman Research Institute, United States of America

## Abstract

Retinal tissue is exceptional because it shows a high level of energy metabolism. Glycogen content represents the only energy reserve in retina, but its levels are limited. Therefore, elucidation of the mechanisms controlling glycogen content in retina will allow us to understand retina response under local energy demands that can occur under normal and pathological conditions. Thus, we studied retina glycogen levels under different experimental conditions and correlated them with glucose-6-phosphate (G-6-P) content and glycogen synthase (GS) activity.

Glycogen and G-6-P content were studied in *ex vivo* retinas from normal, fasted, streptozotocin-treated, and insulin-induced hypoglycemic rats. Expression levels of GS and its phosphorylated form were also analyzed. *Ex vivo* retina from normal rats showed low G-6-P (14±2 pmol/mg protein) and glycogen levels (43±3 nmol glycosyl residues/mg protein), which were increased 6 and 3 times, respectively, in streptozotocin diabetic rats. While no changes in phosphorylated GS levels were observed in any condition tested, a positive correlation was found between G-6-P levels with GS activity and glycogen content. The results indicated that *in vivo*, retina glycogen may act as an immediately accessible energy reserve and that its content was controlled primarily by G-6-P allosteric activation of GS. Therefore, under hypoglycemic situations retina energy supply is strongly compromised and could lead to the alterations observed in type 1 diabetes.

## Introduction

Glucose is the main substrate for energy metabolism in nervous tissue, including retina. Indeed, retina exhibits a high level of energy metabolism requiring constant supply of blood glucose to sustain function [1]. Glucose is phosphorylated to glucose-6-phosphate (G-6-P), which is an important regulatory molecule that enters glycolysis to provide energy via fermentation and subsequent oxidation; after transformation into UDP-glucose, G-6-P can be converted to glycogen. Although the function of glycogen in the nervous system is unknown, there is evidence which suggests that it may act as an energy source during periods of energy deprivation [1]. In the retina, glycogen content fluctuates as the levels of glucose change in the medium [2]. In peripheral organs, glycogen levels are determined by the balance between glycogen synthase (GS) and glycogen phosphorylase (GP), which are the key enzymes for glycogen synthesis and degradation, respectively. GS is regulated by covalent phosphorylation which inhibits the enzyme [3], [4], and by the allosteric activator G-6-P [5], [6], although the relative contribution of these two events in the activation of GS is not well understood. Insulin is thought to promote the dephosphorylation and activation of GS leading to the stimulation of glycogen synthesis [3], [4]. However, in the retina from streptozotocin-diabetic rats, glycogen content and GS activity increased [7]. In diabetes, the retina is exposed to fluctuations in blood glucose concentrations, which may be accompanied by changes in oxygen availability and additional metabolic disturbances. Therefore, we designed in vivo experiments to investigate whether glycogen content in retina is regulated by the GS-allosteric activator G-6-P, or if it might also be regulated by insulin.

## Materials and Methods

### Animals

Adult Long Evans rats (170–200 g) were used in this study. Animals were maintained under standard laboratory conditions (21°C±1, 12 h light dark cycle) and were allowed food and water *ad libitum*. All experiments were conducted between 9:30 and 12:00 am.

All procedures were conducted in accordance with the Mexican Institutes of Health Research (DOF. NOM-062-Z00-1999), the National Institutes of Health Guide for the Care and Use of Laboratory Animals (NIH publication No. 80-23, revised 1996), as well as the Association Research in Vision and Ophthalmology Statement on the Use of Animals in Ophthalmic and Vision Research. The experimental protocol was approved by the Committee on the Ethics of Animal Experiments of our Institution. All efforts were made to minimize animal suffering, and to reduce the number of rats used.

Diabetes was induced by intraperitoneal administration of streptozotocin (65 mg/kg) in 0.05 M citrate buffer, pH 4.5 [8]. Animals were considered diabetic if blood glucose levels were higher than 250 mg/dl (14 mM). Diabetic animals along with age-matched control rats were used after 20 days of streptozotocin administration.

### Insulin administration

Insulin (5 U/Kg; Humulin NPH, Lilly) was administered with a single subcutaneous injection. Blood glucose levels were measured prior to injection of saline (0.9% sodium chloride) or insulin, as well as at 30, 60, 90, and 120 min post injection. At different time points, rats were sacrificed; the eyes were quickly dissected over ice, equatorially hemisected, and for biochemical determinations the retina was peeled away from the optic-cup using fine forceps.

### Glucose administration

Normal rats were fasted overnight for 18 h. Blood glucose levels were measured before and after 30, 60, 90, 120, and 150 min of intraperitoneal administration of glucose (1 g/Kg) or the same volume of saline. Rats were sacrificed approximately 2 h after glucose administration, when blood glucose levels were twice the initial values; retinas were isolated as above for biochemical determinations.

### Biochemical assays

For determination of glucose-6-phosphate, tissues were rapidly homogenized in cold 6% (w/v) perchloric acid. Protein was removed by centrifugation and G-6-P was enzymatically quantified according to Michal, 1984 [9]. The amount of NADPH produced was determined by the absorbance at 340 nm using an extinction coefficient of 6.3.

For glycogen determination, tissues were quickly homogenized with 0.4 ml of ice cold 100 mM NaOH and assayed as described previously [10]. GS and GP activities were measured in tissue homogenates (10% w/v) according to Dringen, 1982 [11]. GS activity was measured in the presence of 6.2 mM of glucose-6-phosphate. Total GP activity was determined by incubating the tissues in the presence of 1 mM AMP [10].

### Western blot analysis

Western blot analyses were performed as previously described [7]. Equal amounts of sample were resolved on a 10% SDS polyacrilamide gel. The proteins were transferred into PVDF Immobilon membranes (Millipore Corp, Billerica, MA). After being blocked with 5% nonfat milk, the membranes were probed with rabbit anti-glycogen synthase 15B1 (1∶500, Cell Signaling Technology, Danvers, MA), or rabbit anti-phospho glycogen synthase (Ser641) (1∶100, Cell Signaling Technology, Danvers, MA), followed by horseradish-peroxidase-conjugated secondary antibody (1∶4000, Amersham Biosciences Piscataway, NJ). Protein loading was normalized to actin using a monoclonal primary antibody (1∶25000, Chemicon, Temecula, CA). The signal was detected by enhanced chemioluminiscence using Chemioluminiscent HRP Substrate (Millipore Corp, Billerica, MA). Densitometry was performed with an Alpha DigiDoc RT (Alpha Innotech, San Leandro, CA) and analysed using a densitometry program (AlphaEase FC Stand Alone; Alpha Innotech, San Leandro, CA).

Protein content was determined using a commercial assay kit (BioRad Lab. Hercules, CA). Blood glucose concentration was determined with a blood glucose monitor (Accu-check, Roche, Indianapolis, IN).

### Statistical analysis

All results are presented as the standard error of the mean ± SEM of at least five separate experiments. Significance was determined by analysis of the variance (ANOVA) using Tukey's post hoc test or Student's t –test, where p<0.05 was taken to indicate statistical significance.

## Results

Previous results indicated that glycogen content in retina fluctuated as the level of glucose changed in the incubation medium [2]. Thus, in order to gain insight into the regulation of glycogen content *in vivo,* we studied glycogen levels and those of the GS- allosteric- activator G-6-P under different conditions.

As shown in Table 1, *ex vivo* retinas from normal rats exhibited low G-6-P (14±2 pmol/mg protein), values that were considerably reduced in the retina from fasted rats (1.8±0.5 pmol/mg protein) (Table 1). In contrast, G-6-P content increased 6 times the control value in the retina from streptozotocin-diabetic rats as well as in fasting rats after glucose administration (Table 1). Confirming a previous report [7], we found glycogen content in retina of diabetic rats increased about 3 times over control levels (Table 1). However, glycogen levels in fasting rats or in fasting rats injected with glucose were similar to those observed in normal rat retina (Table 1).

**Table 1:** Glucose-6-phosphate and glycogen content in the retina of rats under different conditions.

	Glucose-6-P	Glycogen	Blood glucose
**Normal (N)**	14±2	43±4	114±4
**Fasted (F)**	1.8±0.5*	47±8	84±5
**F+glucose**	82±19*	48±5	223±10*
**N+insulin**	2.5±0.1*	48±4	55±6*
**Diabetic**	95±8**	142±11*	478±15*
**D+insulin**	3.7±0.1**	28±3*	60±5*

Normal and 20 day diabetic rats were treated as described in Methods. Normal (N), normal-fasted rats (F) and normal-fasted rats plus glucose administration (F+glucose) were sacrificed 2 h after saline or glucose administration. Normal (N) or diabetic (D) rats were killed after 3 h insulin or saline injection. Glycogen, nmol glycosyl residues/mg protein; glucose-6-P, pmol/mg protein; glucose, mg/dl.

* p<0.01;

** p<0.001 with respect to normal rats.

**Figure 1:**
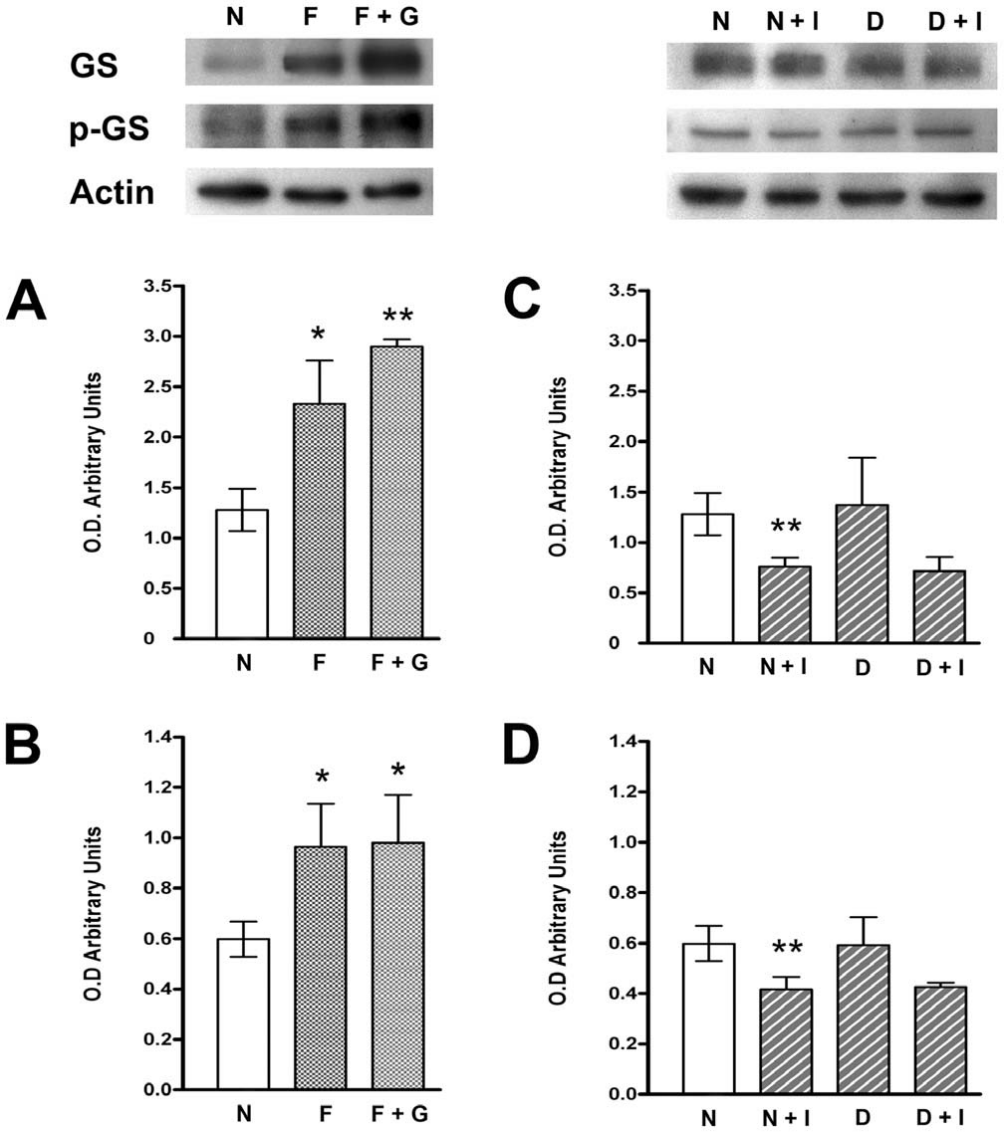
Total and phosphorylated GS in retina. Western blot analysis of representative experiments, top panels. (**A–D**) Graphic representation of the relative levels of total GS (**A, C**) and phospho (Ser 641)-GS (**B, D**). N, normal rat; F, fasted rat; F+G, fasted rat with glucose administration; N+I, normal rat plus insulin administration; D, 20 day streptozotocin-diabetic rat; D+I, diabetic rat plus insulin injection. Values are the mean ± SEM of four experiments. * p<0.05; ** p<0.02.

Because net content of glycogen is regulated simultaneously by both synthesis and breakdown, we determined the activity of GS and GP in the retina of normal and of diabetic rats. The activity of GS was found to be increased (60%) in the retina from streptozotocin-diabetic rats, while no significant changes were observed in the activity of GP (Table 2) [7]. Since GS activity is known to be stimulated by dephosphorylation [3], [4], we determined levels of phosphorylated- GS form (p-GS) under the different conditions tested. The expression levels of total GS (Fig. 1 A) and the p-GS (Fig. 1 B) were significantly increased in the retina of normal fasted rats; however, a similar ratio of p-GS/GS was observed between those of normal and fasted animals.

**Table 2:** Activity of glycogen synthase and glycogen phosphorylase in rat retina.

	Glycogen synthase	Glycogen phosphorylase
**Normal**	1.08±0.06	9.5±0.9
**Diabetic**	1.6±0.12*	10.4±1.5
**Diabetic+insulin**	0.9±0.14	13.6±3.8

Retina from normal, diabetic, and insulin-treated diabetic animals (diabetic+insulin) were processed as described in Methods. Activity of glycogen synthase was measured in the presence of 6.2 mM glucose-6-phosphate. Activity of glycogen phosphorylase was measured in the presence of 1 mM AMP. Activities are expressed as nmol/mg protein/min. Values are the means ± SEM from 5 to 8 rats.

* p<0.01 with respect to the normal rats.

**Figure 2:**
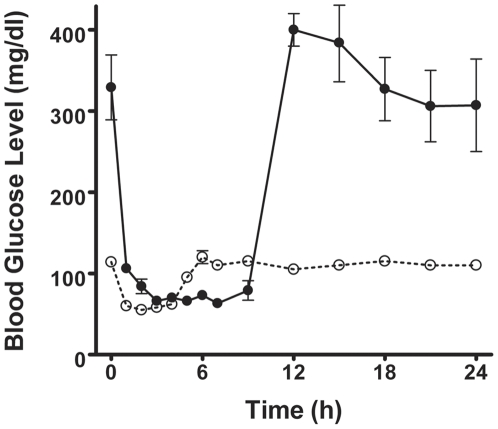
Blood glucose levels following insulin administration. Insulin (5 U/kg) was administered subcutaneously to normal (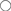) and diabetic rats (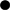). Values are the means ± SEM of at least five different animals. Standard error in normal rats was less than 5%.

The effect of insulin-induced hypoglycemia to induce changes in glycogen levels in retina was also examined. A single subcutaneous administration of insulin (5 U/kg) to normal or to diabetic rats rapidly reduced blood glucose levels. After 1–2 h of insulin administration to normal rats, blood glucose levels diminished about 40% to 50%; they increased reaching normal values about 5 h later (Fig. 2). Similarly, insulin administration to diabetic rats reduced blood glucose levels rapidly, reaching normoglycemic values in 1–2 h, then values declined to 70±6 mg/dl (after 3 h). Afterwards, blood glucose levels increased gradually, and levels of circulating glucose returned to the hyperglycemic state about 12 h later (Fig. 2).

**Figure 3:**
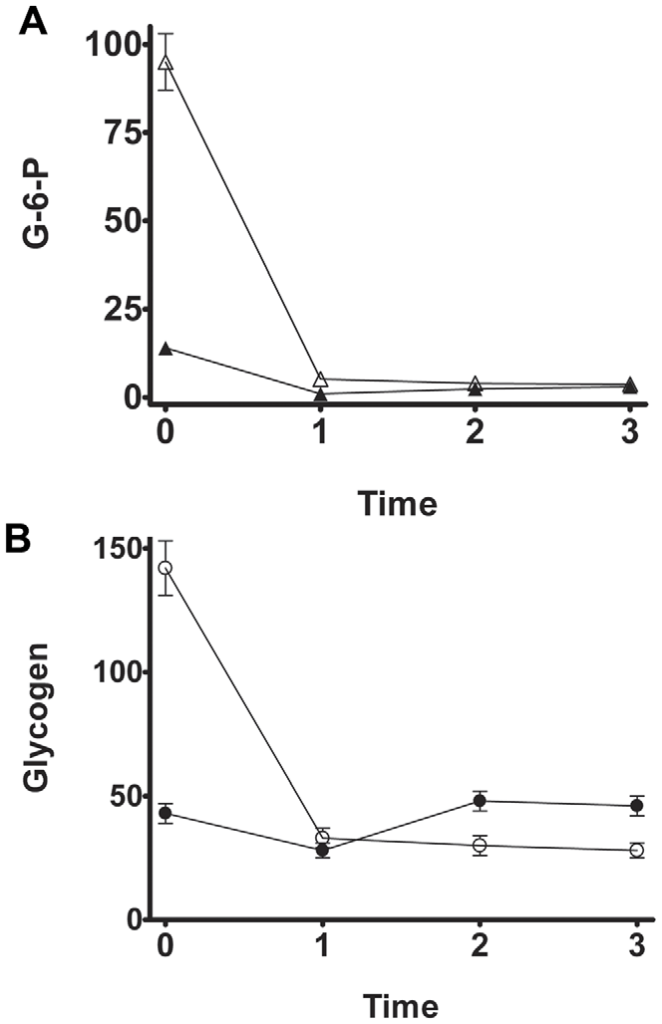
Glucose-6-phosphate lowering patterns and glycogen content in retina following insulin administration. Insulin (5 U/kg) was administered subcutaneously to normal and 20 day diabetic rats. (**A**) G-6-P levels (pmol/mg protein). (**B**) Glycogen content (nmol glycosyl residues/mg protein) in retina. Open symbols, diabetic rats; solid symbols, normal animals. Values are the means ± SEM of at least three different animals.

Following insulin administration, glycogen levels in the retina of normal rats first decreased (30%), then increased rapidly, reaching the normal values 2 h after insulin administration, even though hypoglycemic conditions persisted (55 mg blood glucose/dl) and G-6-P retina content decreased significantly (Fig. 3). In the retina of diabetic rats, glycogen content was 80% reduced after 1 h of insulin injection and remained low 3 h later, time in which considerable low G-6-P levels were observed (Fig. 3). After 24 h insulin administration, glycogen content (86±12 nmol glycosyl residues/mg protein) was significantly lower than that found before insulin administration (142±11 nmol glycosyl residues/mg protein). Moreover, the GS activity was similar to that from normal rats (Table 2).

Furthermore, the expression levels of GS and p-GS were decreased in the retina from normal rats after 2 h insulin administration (Fig. 1 C and D). However, no significant differences between GS or p-GS were observed in the diabetic retina after insulin administration. Moreover, no differences in p-GS/GS ratio were observed between normal or diabetic rat retinas (Fig.1).

**Figure 4:**
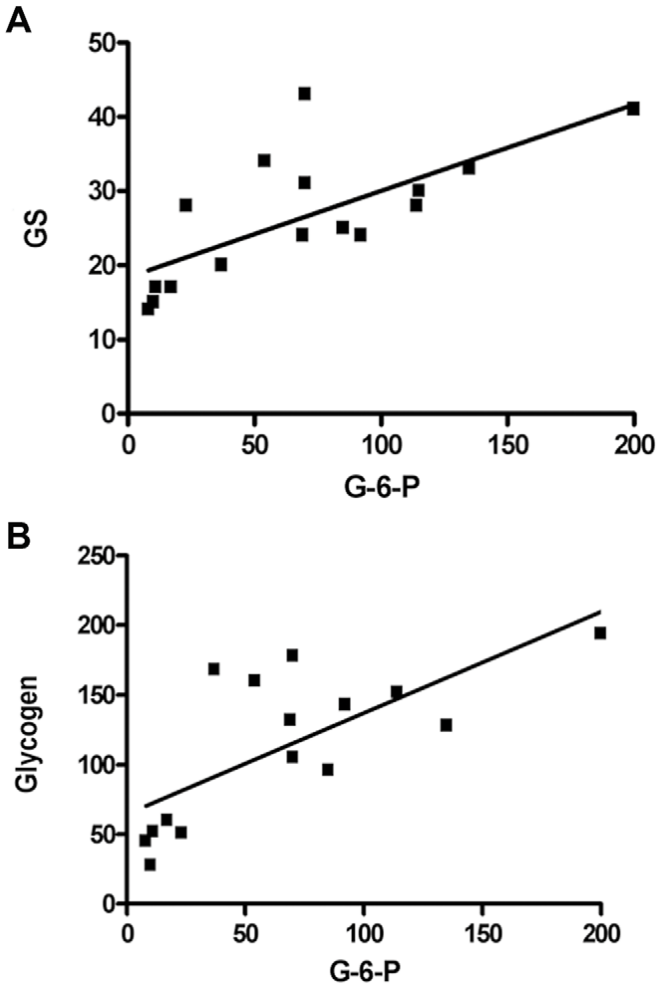
Correlation between glucose-6-phosphate content and glycogen synthase activity in retina. (**A**) Relationship between G-6-P (pmol/mg prot.) and GS activity (nmol/mg prot./min) (r^2^ = 0.51; p = 0.0025). (**B**) Relationship between G-6-P and glycogen content (r^2^ = 0.5; p = 0.0019).

From the present results and the data of an earlier study [7], a significant correlation (r^2^ = 0.5, p = 0.0019) was proved between the G-6-P content and the activity of GS as well as glycogen levels in retina (Fig. 4). Similarly, in agreement with previous results [6], a significant correlation (r^2^ = 0.68, p = 0.0001) was observed between G-6-P and GS activity in liver.

## Discussion

The mammalian retina is a very metabolically active tissue whose energy demands are normally met through the uptake of glucose and oxygen [12], [13]. Glycogen is the only significant energy reservoir in nervous tissue; in retina, glycogen and GP are localized mainly in Muller glial cells [14]. The metabolism of glucose is intimately linked with glycogen levels. Indeed, glycogen content in normal retina was seen to fluctuate with the levels of glucose in the medium [2]. Glycogen content depends on synthesis and degradation. The regulation of GS activity is known to be complex [15], [16], [17]. In peripheral tissues, GS activity is regulated by phosphorylation catalyzed by glycogen synthase kinase-3 (GSK-3) [3], [4], [15]. In addition, G-6-P concentrations allosterically activate GS activity [16], but the relative role of these two events in the regulation of GS activity *in vivo* is largely unknown. In brain, glycogen content is influenced by several neurotransmitters and neuropeptides, including insulin and norepinephrin [18]. In retina, an increase was observed in glycogen content as well as in GS activity in *ex vivo* retinas from streptozotocin-diabetic rats [7]. Therefore, we focused on evaluating the possible mechanisms by which glycogen content in retina is controlled; for this purpose we determined glycogen and G-6-P in the retina of rats under different experimental conditions.

*Ex vivo* retinas from normal rats showed low content of G-6-P which was even lower in rats under fasting conditions or insulin administration. These low levels could indicate a high turnover rate, suggesting G-6-P was rapidly metabolized. In contrast, about 6 times higher levels of G-6-P were found in fasted rats injected with glucose and in hyperglycemic-diabetic rats, suggesting a relationship with the high blood glucose levels found in these animals. Retina glycogen levels were slightly reduced in normal rats treated with insulin, but they were rapidly recovered although hypoglycemic conditions remained. These results imply that glycogen serves as a substantial source of glycosyl units during insulin-induced hypoglycemia and therefore may be neuroprotective [19]. In normal retina, in spite of the remarkable changes observed in G-6-P levels under different experimental conditions, glycogen content remained almost constant, indicating that glucose uptake and hexokinase activity were not limiting. Certainly, the retina shows a high capacity to transport exogenous glucose, since the Glut1 transporter is widely distributed in this tissue [20], [21], [22]. These findings may suggest an important flux of G-6-P to glycogen, suggestive of an insulin role in controlling glycogen content. Although the role of insulin in retina is unknown, this hormone and its receptors have been reported in this tissue [23], [24], [25].

In contrast, high G-6-P and glycogen levels were found in the *ex vivo* retina from diabetic rats. Notably, G-6-P and glycogen levels in diabetic retina were dramatically diminished upon insulin administration and could not be recovered, as occurs in normal rat. These findings suggest differences in G-6-P metabolism between normal and diabetic retina. In reference to this, an interesting finding is that a high lactate retinal content has been reported in diabetic rats [8]; the increase in lactate could result in acidosis which may have deleterious effects.

Besides, in spite of the significant changes observed in glycogen content under the diverse conditions studied, no differences in p-GS levels were found, which suggests that dephosporylation of GS was not an involved mechanism. Consistent with this interpretation, we found a significant correlation of G-6-P levels with GS activity as well as with glycogen content in retina (Fig. 4). Therefore, our results indicated that in retina G-6-P caused allosteric activation of GS, as has been reported in other tissues [16], [26], and that GS activation in turn led to the elevated glycogen content observed in diabetic retina. Thus, we conclude that retina glycogen content served as a glucose reservoir during periods of hypoglycemia and that its levels were regulated by G-6-P concentrations. The high levels of glycogen observed in the streptozotocin–treated rat retina were the result of the hyperglycemia, given that circulating glucose levels are the result of glucose release from liver and its removal from circulation by transport into muscle. The slow recovery of glycogen content in the retina from insulin-treated diabetic rats most likely represent a result of the chronic liver and muscle alterations of glucose disposal reported in type 1 diabetes [27], [28], [29]. Therefore, since retina glycogen serves as an energy reservoir, under the hypoglycemia observed clinically in patients with type-1 diabetes, retinal neuronal function must be seriously compromised. Supporting this statement, glycemic control is a major concern in diabetic patients, particularly those having type 1, who undergo frequent hypoglycemic episodes [30]. Under these conditions, the glycogen content in retina would be about 50% of the normal values and glucose production from glycogen would further support retinal function only for a short time through glycolytic metabolism. Indeed, rabbit and rat retinas incubated in the absence of glucose lead to decline in glycogen [2] and ATP content, and loss of light-induced electrical signals within 30 min [1]. Although it is clear that further studies will be needed to decipher the mechanisms, our results suggest that under the hypoglycemic conditions observed in diabetes, retinal neuronal survival could be compromised. In this respect, it is remarkable that decrease in retinal function was found in chronic hypoglycemia produced in an animal model [31]. Moreover, during the revision of this manuscript, acute hypoglycemia was reported to induce retinal cells death in mouse [32] and significant reduction of central retinal function in humans [33].
